# Impact of drying on the bioactive compounds and antioxidant properties of *bignay* [*Antidesma bunius* (L.) Spreng.] pomace

**DOI:** 10.1186/s43014-022-00122-z

**Published:** 2023-02-07

**Authors:** Claire S. Zubia, Gilda Melanie O. Babaran, Sheba Mae M. Duque, Lotis E. Mopera, Lloyd Earl L. Flandez, Katherine Ann T. Castillo-Israel, Florencio C. Reginio

**Affiliations:** 1 Institute of Food Science and Technology, College of Agriculture and Food Science, University of the Philippines Los Baños, College, 4031 Laguna, Philippines; 2 Science Education Institute – Department of Science and Technology, DOST Compound, General Santos Avenue, 1630 Taguig City, Metro Manila Philippines

**Keywords:** Freeze-drying, Convection oven-drying, Antioxidants, Phenolics, *Bignay*, Pomace

## Abstract

*Bignay* pomace is a processing byproduct that can be a source of bioactive compounds. However, a suitable dehydration method should be considered to efficiently valorize this waste material into high-value food ingredient and maximize its health-promoting properties. *Bignay* pomace was subjected to convection oven-drying and freeze-drying to investigate the effect of these pre-processing techniques on the physicochemical, bioactives, and antioxidant properties of the samples. Both drying methods significantly (*p*<0.05) changed the total phenolic and anthocyanin contents of *bignay* pomace while flavonoids and tannins were not significantly affected. Freeze-drying of samples resulted in higher phenolic content (1742 vs. 1273 mg gallic acid equivalent/100 g DW) and anthocyanin content (496 vs. 223 mg cyanidin-3-glucoside equivalent/100 g DW) than convection oven-drying. Freeze-drying also resulted in higher antioxidant properties based on DPPH, ABTS radical scavenging activity, and FRAP assays. *Bignay* pomace extract was analyzed through HPLC (with photo-diode array detector) for its phenolic profile and nine compounds were identified, with catechin and epicatechin as the dominant components. HPLC analysis also showed that while the drying process does not influence the phenolic profile of the samples, it significantly affected the concentration of phenolic compounds present. The results of this study showed that freeze-drying is a more viable method to retain the majority of *bignay* pomace’s functional properties compared to convection oven-drying.

**Graphical Abstract:**

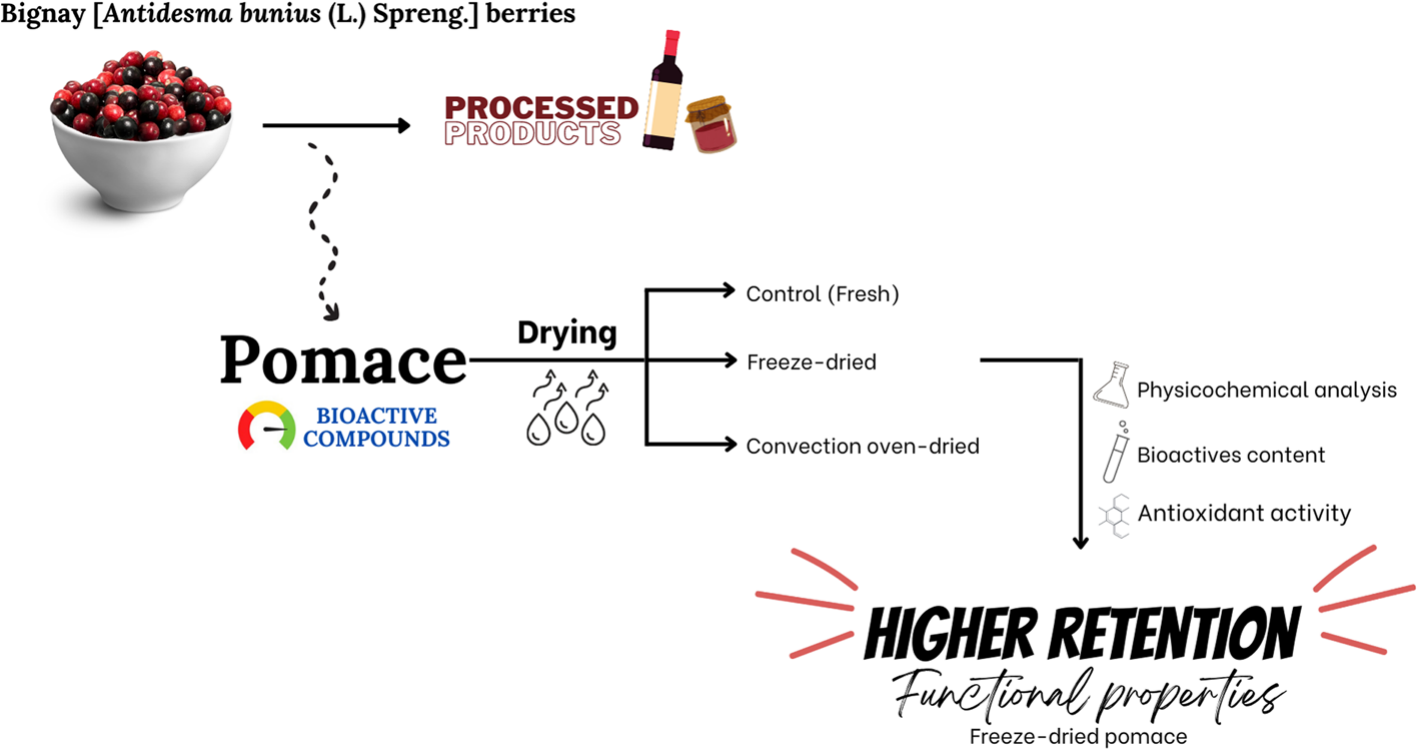

## Introduction

The processing of horticultural crops produces a considerable amount of waste materials, which is mainly composed of biodegradable organic matter. Their disposal can pose serious health and environmental problems. These byproducts are commonly disposed in landfills but their decomposition can favor the proliferation of pathogenic microorganisms or produce toxic leachates (Palaniveloo et al. 2020). To minimize associated environmental impacts and the cost of material disposal, researchers are focusing on finding ways to recover, recycle, and upgrade these residues into valuable materials (Capanoglu et al. 2022). Byproducts of plant origin such as pomace could be an inexpensive source of some beneficial bioactive compounds, which can be used in pharmaceutical and food industries (Iqbal et al. 2021).

Tropical fruit wine is a growing industry in the Philippines, particularly in the northern region. *Bignay* [*Antidesma bunius* (L.) Spreng.] is one the most commonly used substrate due to its popularity among consumers. *Bignay* is an indigenous berry, which owes its popularity to the increasing awareness of the population to consume food that can confer certain health benefits. The perceived consumer health benefits of *bignay* and its food products could be linked to reports of its phytochemical contents and antioxidant properties (Butkhup & Samappito 2008; Castillo-Israel et al. 2020; Hardinasinta et al. 2020; Islary et al. 2017; Jorjong et al. 2015; Shariful Islam et al. 2018). *Bignay* wine is prepared from fully ripe *bignay* fruits, of which the pomace is the byproduct after the whole fruit is passed through a pulper machine. The pomace consists mainly of seeds, skin, and some pulp. Because of its high level of anthocyanin, a prominent phenolic in red and dark berries, *bignay* pomace exhibits dark purple color (Butkhup & Samappito 2008).

*Bignay* fruit as a whole is a good source of bioactive materials. These bioactive compounds are primarily phenolics, which have been reported to exhibit biological activities that include antioxidant, anti-inflammatory, antiproliferative, and antimicrobial properties and have been associated with reduced risk of cancer and cardiovascular diseases (Albuquerque et al. 2021; Hardinasinta et al. 2020; Tao et al. 2014). Besides the beneficial health effects of *bignay*, its application in food is also important as it contributes to food flavor, color, and stability. Phenolic compounds and/or their derivatives are added to a variety of food products to enhance their nutritional value or to influence the functional properties of biomolecules (Ebrahimi & Lante 2021). With these findings, *bignay* pomace could be a potential source of bioactive materials.

*Bignay* pomace, in its fresh, unprocessed form, is highly perishable due to its high moisture content and water activity. To prolong the shelf life of pomace and to facilitate efficient handling and storage, dehydration process is usually taken as an initial step prior to any other processing methods. Dehydration reduces moisture to low levels making it less available for microbial growth and enzymatic reactions. Different dehydration techniques can be employed, but the appropriate method should be able to preserve its overall quality. It is necessary to select a preservation method for *bignay* pomace that can minimally affect its nutritional and functional properties, on top of cost efficiency, to be able to integrate it as a functional food ingredient in the future. Certain drying techniques had been studied and found to preserve the biologically active compounds in grape pomace (Sokač et al. 2022; Tseng & Zhao 2012). Some of these techniques could also be effective methods for *bignay* pomace.

With the primary objective of preserving the functional properties of *bignay* pomace, this study investigated the effects of two common dehydration methods (convection oven-drying and freeze-drying) on the quality of *bignay* pomace. The impact of drying was assessed based on proximate composition, physico-chemical properties, bioactive components, and antioxidant properties.

Moreover, before the bioactive materials in *bignay* pomace can be efficiently exploited for different food applications, there is a need to establish that sufficient quantities are present in this byproduct. To date, there is limited data on the bioactive compounds and antioxidant properties of *bignay* pomace. Results of this research will provide baseline information that is needed to assess the potential of *bignay* pomace as a food ingredient to create healthier food alternatives from a food processing waste product.

This study was done on a laboratory-scale but the promising results could prompt more research and pilot-scale studies that can eventually be elevated to industrial/commercial levels. Currently, more than 50% of wine processors in the country are utilizing *bignay* as a raw material (Department of Trade and IndustryTI-Region 2, personal communication, August 13, 2021). The Global Agricultural Information Network (2018) reported that Philippines had a steadily progressing wine market until it was hit by the coronavirus pandemic. With this increasing market, it is likely that there will be an increase in the local production volume, which will generate more waste products. Processing the large volumes of byproducts will be made easier with the availability of large-scale convection oven- and freeze-dryers on the market.

## Materials and methods

### Raw materials

Fully ripened *bignay* berries (purple to black color) were harvested in Los Baños, Laguna, Philippines. The harvested berries were washed with clean running water to remove any dirt and the excess water was allowed to drain in a colander. The fruits were then loaded into the pulper machine to separate the pulp from the seed and skin (pomace). The pomace was then manually mixed, portioned in polyethylene bags at 1 kg per pack, and kept frozen at -20 °C until further processing. All samples were ground prior to analysis using a grinder (TGS-CG9100, Deli Chef, China) and passed through a 20-mesh sieve.

### Chemicals

Analytical grade chemicals and reagents were used in the analyses of samples. All phenolic standards used are from Sigma-Aldrich (Singapore).

### Drying treatments

Frozen unprocessed pomace was thawed at room temperature prior to processing. After thawing, the sample was divided into three lots, two of which were immediately subjected to two drying treatments: (1) convection oven-drying and (2) freeze-drying in a freeze-dryer built by GECAR Machine Solution, Inc. (Philippines). The parameters used in these methods were determined from a preliminary experiment to achieve a final moisture content and water activity of below 10% and 0.6, respectively.

Pomace for convection oven-drying was first spread thinly on a 74 cm × 51.5 cm × 2.5 cm stainless steel tray. The trays were then loaded in an oven-dryer manufactured by TEW Electric Heating Equipment Co. Ltd. (Taiwan). The samples were then dried at at 45 °C for 48 hours.

For samples for freeze-drying, the pomace was placed on a 50 cm × 32 cm × 2 cm stainless steel tray. Freeze-drying was carried out at a heater temperature of 25–30 °C, chilling temperature of -30 °C and operating vacuum pressure of 100–300 Pa for 30 hours. The processed samples were manually mixed, vacuum-packed using a portable vacuum food sealer at -60 kPa pressure, and stored in the freezer until further analysis. Frozen unprocessed pomace (thawed after freezing at -20 °C) was used as control/reference and was referred to as a “fresh” sample.

### Physico-chemical analysis

#### Proximate composition

Analysis of the proximate composition of samples was conducted according to the Association of Official Analytical Chemists (AOAC 2019) procedures to determine the moisture, ash, crude fat, crude protein, and crude fiber contents of oven- and freeze-dried samples.

#### Water activity

The water activity of samples was determined using a water activity meter (LabSwift-aw, Novasina, Lachen, Switzerland) following manufacturer’s instruction manual (Novasina 2010). Approximately 2 g of ground pomace samples were evenly placed on the sample holder for measurement.

#### Instrumental color

The color profile of pomace samples was determined using a handheld chromameter (CR-400 Chroma Meter, Konica Minolta, Japan) based on the method used by Moritsuka et al. (2019) with modifications. Approximately 6 g of homogenized samples were firmly packed in a 7 cm × 6 cm polypropylene bag before measurement. Data were expressed according to the CIELAB system where L* indicates brightness/luminosity, a* for redness (+) to greenness (−), and b* for yellowness (+) to blueness (−). From the L*, a*, and b* values obtained, other color parameters such as the chroma, C* (Eq. 1), hue angle, H° (Eq. 2), and color difference, ΔE* (Eq. 3) were calculated:

1 $${C}^{\ast }=\sqrt{a^{\ast 2}+{b}^{\ast 2}}$$

2 $$H{}^{\circ}={\mathit{\tan}}^{-1}\frac{b}{a}$$

3 $$\Delta {E}^{\ast}=\sqrt{{\left(\Delta {L}^{\ast}\right)}^2+{\left(\Delta {a}^{\ast}\right)}^2+{\left(\Delta {b}^{\ast}\right)}^2}$$

where ΔL*, Δa* and Δb* represent the change in the color coordinates compared to the sample before drying. The values were automatically computed and recorded by the chromameter.

### Preparation of extracts

A preliminary screening was conducted to determine the extraction protocol and the method with the highest total phenolic content in the sample was selected. The final protocol used for the extraction of bioactives from *bignay* pomace was based on the method of Castillo-Israel et al. (2020) with some modifications. It was carried out by mixing 0.5 g of ground sample (passed through a 20-mesh sieve) with 15 mL of 50:50:1 (v/v/w) solution of absolute ethanol: distilled water: citric acid. The mixture was then incubated (Biobase BOV-D50, Biobase Meihua Trading Co., Ltd., China) at 60 °C for 60 min, subjected to sonication (Ultrasonic Cleaner 8892, Cole-Parmer, U.S.A.) for 15 min, and centrifuged at 70 × *g* for 15 min. The supernatant was filtered through Whatman® No.1 filter paper (Sigma-Aldrich, U.S.A.) and the filtrate was collected, divided into 2.5 to 4 mL portions, and stored at -20 °C until further analysis. The residue after the centrifugation process underwent the same extraction and filtration process twice.

### Quantification of bioactive compounds

The quantification of total phenolics, total flavonoid, total monomeric anthocyanin, and condensed tannins was done through spectrophotometric methods using Shimadzu UV 1900 (Shimadzu Corp., Japan).

#### Total Phenolics Content (TPC)

The total phenolic content was analyzed according to the ISO (International Organization for Standardization, 2005) 14,502–1 with minor modifications, using the Folin-Ciocalteu method. Approximately 0.30 mL sample extract was mixed with 1.5 mL 10% (v/v) Folin-Ciocalteu reagent and 1.2 mL 7.5% (w/v) sodium carbonate. The mixture was then left for 60 min in the dark at room temperature. The absorbance was then measured at 765 nm. Blank was prepared by replacing the samples with distilled water and adding Folin-Ciocalteu reagent and sodium carbonate. The standard used for the calibration curve was gallic acid. The total phenolic content of the samples was expressed in milligrams gallic acid equivalent per 100 g of sample in dry weight (mg GAE/100 g DW).

#### Total Flavonoid Content (TFC)

The total flavonoid content was evaluated according to Fattahi et al. (2014). One hundred (100) μL of extract was mixed with 4 mL distilled water and added with 0.3 mL 5% (w/v) sodium nitrite. After 5 min, 0.3 mL 10% (w/v) aluminum chloride was added and the solution was allowed to stand for 6 min. After which, 2 mL of 1 M sodium hydroxide was added, diluted with 3.3 mL distilled water, and mixed thoroughly. The absorbance was determined at 510 nm against the blank. Catechin was used as standard for the calibration curve. The total flavonoids content of the extract was expressed as mg catechin equivalents (CE) per 100 g of sample in dry weight (mg CE/100 g DW).

#### Total Monomeric Anthocyanin content (TMA)

The total monomeric anthocyanin content was analyzed using the pH differential method (AOAC method 2005.02). The appropriate dilution factor was initially determined by diluting the sample with pH 1.0 buffer (potassium chloride, 0.025 M) to have an absorbance between 0.2 and 1.4 AU at 520 nm. Based on the determined dilution factor, 2 dilutions were prepared: (1) with potassium chloride buffer (0.025 M, pH 1.0) and (2) with sodium acetate buffer (0.40 M, pH 4.5). The absorbance was measured at both 520 and 700 nm within 20–50 min of preparation against distilled water as blank. The total monomeric anthocyanin content (mg C-3-GE/L extract) was calculated using Eq. 4.

4 $$Total\ monomeric\ anthocyanin\kern0.5em \Big( mg\ cyanidin-3-glucoside\ equivalents/L\Big)=\frac{A\times MW\times DF\times {10}^3}{\varepsilon \times l}$$

Where:

A = (A_520nm_ – A_700nm_)_pH 1.0_ - (A_520nm_ – A_700nm_)_pH 4.5_

MW = molecular weight of cyanidin-3-glucoside (449.2 g/mol)

DF = dilution factor

ε = molar extinction coefficient of cyanidin 3-glucoside (26,900 L mol^− 1^·cm^− 1^)

l = = cell path length (1 cm)

10^3^ = factor conversion from g to mg

Total monomeric anthocyanin content in mg cyanidin-3-glucoside equivalents/L was then converted into mg cyanidin-3-glucoside equivalents for every 100 g of sample in dry weight (mg/100 g DW)

#### Condensed Tannins Content (CTC)

Condensed tannins content was determined according to Medini et al. (2014) using the vanillin-HCl method. The diluted extract (0.05 mL) was mixed with 3 mL 4% (w/v) vanillin solution in methanol and 1.5 mL of concentrated HCl. The solution was allowed to stand for 15 min in the fume hood before measurement of its absorbance at 500 nm versus absolute methanol as blank. The results were expressed as mg catechin equivalent per 100 g dry weight of the sample (mg CE/g DW).

### Antioxidant properties

#### Ferric Reducing Antioxidant Power (FRAP)

The determination of FRAP was carried out according to Castillo-Israel et al. (2020) and Tomasina et al. (2012). FRAP reagent was freshly prepared by mixing 300 mM acetate buffer (pH 3.6), 10 mM TPTZ solution, and 20 mM ferric chloride in 10:1:1 (v/v/v) ratio. The solution was placed in an incubator (Biobase BOV-D50, Biobase Meihua Trading Co., Ltd., China) at 37 °C for 30 min. The assay was conducted by mixing 2.7 mL FRAP reagent with 0.3 mL of appropriately diluted sample, incubated at 37 °C for 5 min, and read at 620 nm for its absorbance. The results were reported as mg Trolox equivalents for every 100 g sample in dry weight (mg TE/100 g DW).

#### 2,2-Diphenyl-1-1picrylhydrazyl (DPPH) radical scavenging activity

The DPPH scavenging activity was determined according to Marinova and Batchvarov (2011) with minor modifications. The 1.5 mL diluted extract was mixed with 1.5 mL 60 μM methanolic solution of DPPH. The solution was mixed well and allowed to stand for 30 min under dark conditions. The absorbance was measured at 517 nm versus absolute methanol as blank. The DPPH scavenging activity was expressed as mg Trolox equivalent (TE) per 100 g sample in dried weight (mg TE/100 g DW).

#### 2,2′-azino-bis(3-ethylbenzothiazoline-6-sulfonic acid) (ABTS) radical scavenging activity

ABTS scavenging activity was determined according to Castillo-Israel et al. (2020) and Tomasina et al. (2012). A 7 mM ABTS^●+^ and 2.45 mM potassium persulfate solution in a 1:1 ratio was prepared and allowed to stand for 16 hours in a dark room. The solution was diluted with 50% (v/v) ethanol to obtain the absorbance of 0.70 ± 0.02 at 734 nm. The assay was conducted by mixing 0.3 mL of the diluted extract with 2.7 mL ABTS solution, incubated in the dark for 15 min, and read at 734 nm for its absorbance. The result was expressed as mg Trolox equivalent (TE) per 100 g sample on a dry basis.

### Identification and quantification of specific phenolic compounds by HPLC

Identification and quantification of the phenolic compounds present in *bignay* pomace were done by high-performance liquid chromatography equipped with a photo-diode array detector based on the method described by Butkhup and Samappito (2008) with modifications. Phenolic standard solutions and freshly prepared sample extracts were injected into the Shimadzu Prominence HPLC system (Shimadzu Corp., Japan) by an autoinjector at an injection volume of 20 μL. Before analysis, the standard solutions and sample extracts were filtered through a 0.22 μm PVDF filter (Labfil, Zhejiang Alwsci Technologies Co., Ltd., China). Chromatographic separation of the compounds was carried out using Inertsil ODS-3 chromatography column (250 mm × 4.5 mm i.d., 5 μm particle size) (GL Sciences, Inc., Japan), using gradient mode of elution. The mobile phase was a mixture of solvent A (acetonitrile:water acidified with acetic acid; 2:98 v/v) and solvent B (acetonitrile:water acidified with acetic acid; 98:2 (v/v). For a total run time of 40 min, the gradient of solvents was programmed as follows: 0–3 min, 10% B; 3–8 min, 20% B; 8–12 min, 30% B; 12–13 min, 40% B; 13–20 min, 50% B; 17–20 min, 50% B; 20–30 min, 70% B; 30–35 min, 90% B; 35–40 min, 10% B. Elution was carried out at 0.8 mL/min flow rate, with column temperature maintained at 30 °C.

The phenolic compounds in the sample were compared with the relative retention times of the standards used (gallic acid, catechin, epicatechin, rutin hydrate, caffeic acid, syringic acid, ellagic acid, p-coumaric acid, trans-ferulic acid, myricetin, resveratrol, and quercetin). For quantitative analysis, the concentration of each compound in the sample was calculated based on the calibration curve (R^2^ ≥ 0.99) obtained by plotting the peak area measurements obtained at different concentrations.

### Statistical analysis

Each analysis was conducted with at least three independent replicates and the results were expressed as mean ± standard deviation. Data were subjected to Levene’s test for homogeneity of variance and analysis of variance (ANOVA) (α = 0.05). Brown-Forsythe and Welch tests were used to determine significant differences among the means for data sets that violated the assumption of homogeneity of variance. Tukey’s Honestly Significant Difference (HSD) test was carried out as post-hoc analysis at 95% level of confidence. Outliers were determined using Dixon’s Q-test. All statistical analyses were performed using IBM SPSS Statistics for Windows, Version 27.0 (IBM Corp., New York).

## Results and discussion

Different parameters were evaluated to investigate the impact of two drying methods (convection oven-drying and freeze-drying) on the properties of *bignay* pomace. These include proximate composition, physico-chemical properties, bioactive components, and antioxidant properties. These parameters were assessed to determine the most appropriate dehydration method for *bignay* pomace to become a shelf-stable source of bioactive materials.

### Physicochemical profile of *bignay* pomace as affected by drying techniques

#### Proximate composition

Table 1 shows the physico-chemical profile of *bignay* pomace subjected to different drying methods. The initial moisture content of the fresh sample was very high with more than 50% but it was significantly reduced after drying. To inhibit the growth of microorganisms, the moisture content must be below 10% (Vera Zambrano et al. 2019), and this was achieved with both oven-drying and freeze-drying. Between the two drying methods, freeze-drying had lower moisture content, however, the difference was not significant (*p*<0.05). This shows that the parameters employed in both drying techniques are equally effective in decreasing the moisture content of pomace.

**Table 1:** Proximate composition and physico-chemical profile of *bignay* pomace as affected by drying techniques

Parameter	Treatment^1^		
	Fresh	Convection oven-dried	Freeze-dried
Moisture content^2^	52.25 ± 1.10^a^	7.59 ± 0.20^b^	6.09 ± 0.27^b^
Ash^3^	6.12 ± 0.31^a^	6.03 ± 0.27^a^	6.23 ± 0.12^a^
Crude protein^3^	8.23 ± 0.52^a^	8.41 ± 0.76^a^	7.88 ± 0.29^a^
Crude fat^3^	7.50 ± 0.21^a^	2.10 ± 0.12^c^	4.36 ± 0.26^b^
Crude fiber^3^	45.79 ± 4.22^a^	41.08 ± 1.23^a^	43.17 ± 1.10^a^
Water activity^2^	0.77 ± 0.01^a^	0.39 ± 0.01^b^	0.30 ± 0.00^c^
Color analysis			
L*	38.53 ± 0.05^c^	41.45 ± 0.45^a^	39.52 ± 0.28^b^
a*	8.66 ± 0.26^b^	8.30 ± 0.13^b^	10.64 ± 0.16^a^
b*	5.26 ± 0.36^a^	3.12 ± 0.06^b^	1.37 ± 0.08^c^
Chroma (C*)	10.13 ± 0.41^a^	8.86 ± 0.13^b^	10.72 ± 0.17^a^
Hue angle (H°)	31.27 ± 1.00^a^	20.61 ± 0.34^b^	7.35 ± 0.34^c^
ΔE*		3.67 ± 0.36	4.49 ± 0.22

^1^Values are expressed as mean + standard deviation (*n* = 3). Means with different superscripts (a-c) within a row, indicate significant differences (*p*<0.05) using Tukey’s HSD test

^2^Values are expressed as percent fresh basis (%fb)

^3^Values are expressed as percent dry basis (%db)

On a dry basis, *bignay* pomace was found to consist mainly of fiber, while protein, fat, and ash were detected in smaller amounts. These components, except the crude fat content, were not significantly affected by both drying methods. While it is interesting to note that crude fat content was found to be lower in both oven- and freeze-dried samples compared to fresh *bignay* pomace, a similar observation was reported by Singhal et al. (2022). This decrease in the crude fat content may be attributed to the oxidation of fats, which produces various volatile compounds (Qu et al. 2015).

#### Water activity

Water activity (Aw) was measured to partially assess the stability of samples after the drying process. The initial Aw of the sample was significantly reduced by 49 and 61% after oven-drying and freeze-drying, respectively. The final water activity of the dried samples were around 0.3. This makes the pomace more stable based on the inhibition of microbial growth (< 0.60) and enzymatic activity (< 0.75) (Prabhakar & Mallika 2014; Tapia et al. 2020).

#### Color analysis

**Fig. 1:**
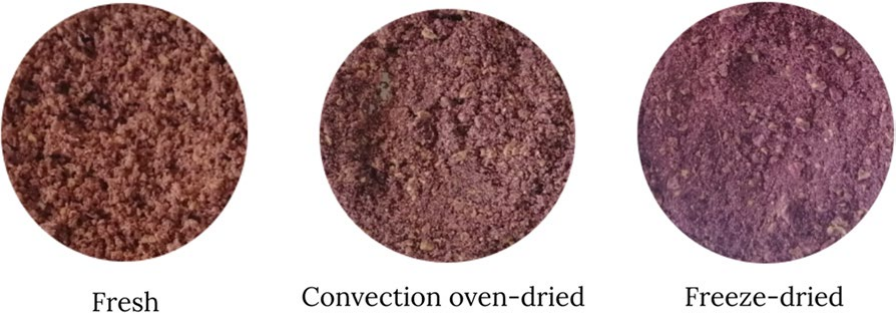
Ground and sieved *bignay* pomace samples

Drying resulted in visually perceptible color changes in the samples (Fig. 1). It is notable that freeze-dried pomace exhibited a purple color, which is generally associated with positive consumer perception when applied to food since natural and bright colors creates an impression of high-quality, healthy, and nutritious food (Amaya & Nickell 2015). On the other hand, fresh and convection oven-dried pomace displayed a yellowish color. These discernible differences in the visually observed colors correspond to the obtained colorimetric values of the samples, which showed significant differences.

The L* values show that the brightness/luminosity significantly increased with drying. Convection oven-dried pomace exhibited the highest L* value, which corresponds to a brighter product color. On the other hand, freeze-dried pomace exhibited the highest a* value, which translates to a more reddish color than the rest of the samples indicating that more of the coloring compounds present in the sample were retained during the process. In terms of b*, fresh sample had the highest value while freeze-dried pomace exhibited the lowest value. Increasing positive b* values indicate increasing yellowness in color, which was manifested visually in the fresh sample that appeared to be more yellowish (Fig. 1).

The computed chroma (C*), hue angle (H°), and color difference (ΔE*) of samples showed the significant effect of drying in instrumental color. Chroma is considered the quantitative attribute for colorfulness or the strength of a color. The higher the C* value, the greater is the color saturation. Amongst the samples, freeze-dried pomace exhibited the highest C* value, which translates to high color intensity that is perceived (Pathare et al. 2013). For H°, the highest and lowest values were observed in fresh and freeze-dried samples, respectively. This data obtained indicates proximity of the samples to the red color spectrum. Visually, freeze-dried sample has a darker color compared to the fresh and convection oven-dried pomace. Graphically, an H° value of 0° represents a red hue, 90° to yellow, 180° to green and 270° is attributed to blue (Santos et al. 2020). For ΔE*, results showed that the convection oven-dried pomace had a lower value compared to the freeze-dried sample, however the differences are not significant. A ΔE* value of 1.0 or lower denotes a non-perceivable color difference (Karma 2020). In the case of *bignay* pomace samples, the values obtained signify a perceptible difference in the color of the dried pomace with respect to the reference (fresh pomace). This conforms to what is visually perceived in the samples. It can be noted in Fig. 1 that the color of convection oven-dried sample and the fresh sample are more similar whereas the freeze-dried pomace exhibited a darker color.

### Effect of drying techniques on the bioactive compounds in *bignay* pomace

Table 2 shows the impact of convection oven-drying and freeze-drying on the total phenolic content (TPCA), total monomeric anthocyanin content, and total flavonoid and condensed tannins content. Data shows significant changes (*p*<0.05) in the total phenolic content and total anthocyanin content of fresh *bignay* pomace after drying. The concentration of total phenolics after undergoing freeze-drying and convection oven-drying decreased. Between the two drying methods, a higher total phenolic content in the pomace was observed after freeze-drying compared to convection oven-drying. From an initial amount of 1967.32 mg GAE/ 100 g DW in fresh pomace, the TPC changed to 1767.48 mg GAE/ 100 g DW after freeze-drying, which is equivalent to a reduction of less than 1%. A much lower concentration of total phenolics was observed in convection oven-dried pomace at 1284.45 mg GAE/ 100 g DW accounting for a 35% loss.

**Table 2:** Phytochemical components of *bignay* pomace as affected by drying techniques

Treatment	Total Phenolic Content (mg GAE/100 g DW)	Total Monomeric Anthocyanin Content (mg C3GE/100 g DW)	Total Flavonoid Content (mg CE/100 g DW)	Total Condensed Tannins (mg CE/100 g DW)
**Fresh**	1967.32 ± 18.90^a^	171.95 ± 1.42^c^	3364.11 ± 215.01^a^	1382.06 ± 143.34^a^
**Convection oven-dried**	1284.45 ± 4.09^c^	210.97 ± 6.39^b^	3085.55 ± 242.96^a^	1441.54 ± 161.97^a^
**Freeze-dried**	1767.48 ± 12.08^b^	474.89 ± 6.41a	3452.72 ± 165.66^a^	1702.45 ± 191.29^a^

Values are expressed as mean ± standard deviation (*n* = 3). Means with different superscripts (a-c) within a column, indicate significant differences (*p*<0.05) based on ANOVA and Tukey’s HSD test

Literature available investigating the effect of drying methods on the bioactive components of *bignay* is limited to none, and the same holds true for *bignay* pomace. However, there are some studies done on other bioactive-rich plant materials that can be used to compare the results of this study. Different researchers have distinct findings depending on the drying method employed. Drying may lead to degradation of phenolic components because their structures are easily destroyed when subjected or exposed to heat, oxygen and freezing (Tan et al. 2019). This explains the reduction in the TPC of *bignay* pomace in both drying techniques. With respect to convection oven-drying and freeze-drying, this study conforms with the findings as Orphanides et al. (2013) where it was reported that freeze-drying resulted in higher total phenolic content in spearmint compared to convection oven-drying. Likewise, Kumar et al. (2022) observed freeze-drying to be a more effective technique than convection oven-drying when applied to *Citrus sinensis* L. Osbeck. Freeze-drying resulted in higher retention of total phenolic contents over convection oven-drying because it is performed at lower temperature. This minimized the reduction in the amount of total phenolic contents since the stability of polyphenols is greatly affected by the temperature, which can cause chemical and enzymatic degradation (Akowuah et al. 2009; Mphahlele et al. 2016). The exposure to oxygen and heat during convection oven-drying may have favored the activity of polyphenol oxidase leading to degradation of phenolic compounds (Çoklar & Akbulut 2017).

Freeze-drying can increase or decrease the total phenolic content depending on the plant material, among other factors. Gümüşay and Yalçın (2019) reported an increase in the total phenolic content of kiwi fruit and cherry laurel. Similar findings were also observed in jujube (Gao et al., 2012), blueberry (Kwok et al. 2004), and stinging nettle leaves (Garcìa et al. 2021). On the other hand, phenolic contents of pomelo peels (Rahman et al. 2018), mango, papaya, and watermelon (Shofian et al. 2011), pineapple (Izli et al. 2018), black grape (Çoklar & Akbulut 2017), bilberry and raspberry (Michalczyk et al. 2009) have decreased after freeze-drying. The same observation was noted in this study where the TPC in *bignay* pomace decreased after freeze-drying. This decrease in the total phenolic content could be attributed to certain phenolic constituents that might have been degraded during freeze-drying. Possible reason for this is the disruption of cell membrane from the formation of ice crystal, which could have promoted enzyme activity (Seke et al. 2021). Tan et al. (2019) attributed the lower phenolic content observed in freeze-dried litchi compared to fresh litchi fruits to the rapid change in pressure during freeze-drying, which may have caused the degradation of some phenolic components.

On the contrary, the total monomeric anthocyanin content of *bignay* pomace was positively affected by the drying methods employed. The amount of anthocyanin analyzed from convection oven-dried and freeze-dried samples were significantly higher than in the unprocessed pomace. From the initial value of 171.95 mg C3GE/100 g DW, the anthocyanin content of *bignay* pomace remarkably increased by 176% after freeze-drying (474.89 mg C3GE/100 g DW), while in convection oven-drying the increase was at 23% (210.97 mg C3GE/100 g DW).

The findings of Michalczyk et al. (2009) on strawberry is consistent with the trend observed for *bignay* pomace where freeze-drying increased the total anthocyanin content. Çoklar and Akbulut (2017) also observed higher individual anthocyanins in the freeze-dried grape than in the fresh sample. During drying process, there are several mechanisms that can impart positive or negative effect on the bioactive components of a plant material. While cell disruption can cause the degradation of certain phenolics, it can also help facilitate a more efficient extraction of cellular component by allowing better solvent infiltration (Sablani et al. 2011). In freeze-drying, cell-disruption is brought by the formation of ice crystals. With this, the cellular matrix is no longer intact and thus, allows some bound cellular components to be freely released during extraction (Mphahlele et al. 2016). In addition to this, freeze-dried materials have greater porosity (80 to 90% than those produced by other drying methods such as convective-, microwave-, and vacuum-drying methods), which allowed higher extraction efficiency (Çoklar & Akbulut 2017).

Interestingly, the total flavonoid content (TFC) and condensed tannins content (CTC) of *bignay* pomace were found to be not significantly affected by both freeze-drying and convection oven-drying. Likewise, the TFC and CTC of freeze-dried and convection oven-dried samples were found to be not significantly different. Elshaafi et al. (2020) reported similar findings on fig (*Ficus carica* L.) BTM 6 cultivar leaves. Samples that were subjected to freeze-drying and oven-drying (60 °C) showed no significant difference on their total flavonoid contents. A study on the thermal stability of flavonoids (rutin, naringin, eriodictyol, mesquitol, luteolin, and luteolin 7-O -glucoside) by Chaaban et al. (2017) demonstrated that most of the analyzed compounds can remain stable even at higher temperature. Rutin, eriodyctiol, and luteolin 7-O-glucoside showed a decrease of less than or equal to 10% after 2 h of treatment at 70 °C and no degradation was observed for naringin at temperatures below 100 °C. The temperature employed for oven-drying of *bignay* pomace was 40 °C, and this may not be enough to cause significant degradation on its flavonoid components.

For condensed tannins content, Larrauri et al. (1997) similarly observed no significant difference between the freeze-dried and oven-dried (60 °C) red grape pomace peels. This may be attributed to the stable nature of tannins. Latos-Brozio and Masek (2020) reported that tannins are more thermally stable compared to other phenolic compounds because of its complex aromatic structure. Tannins are also more stable because of their ability to bind to protein and fibers (Larrauri et al. 1997).

In this study, it was also established that *bignay* pomace contain fairly high concentrations of phenolics, anthocyanin, and flavonoids and are comparable to the results obtained by Butkhup and Samappito (2008) on whole *bignay* fruit. The study evaluated methanolic extracts of the whole *bignay* fruit from different cultivars and found that total anthocyanin ranged from 3.34 to 1414.94 mg C3GE /100 g DW and TPC was from 547 to 1321 mg GAE/ 100 g DW while total flavonoid content was from 114.67 mg /100 g FW to 397.90 mg/100 mg FW (Butkhup & Samappito 2008). *Bignay* pomace was also found to have almost the same concentration of total phenolics as the pulp of *bignay* fruit that were grown in the Philippines. In terms of total flavonoid content, *bignay* pomace turned out to be more superior than the flesh. Fresh *bignay* pomace had a TPC of 1967.32 mg GAE/100 g DW and TFC of 3364.11 mg CE/100 g DW. The methanolic extract of the flesh in the study of Barcelo et al. (2016) had a TPC of 1978 mg GAE/100 g DW and TFC of 1526.7 mg QE/100 g DW. Recuenco et al. (2016) also analyzed the methanolic extract of the flesh and recorded a TPC of 1839 mg GAE/100 g DW and TFC of 404 mg CE/100 g DW.

### Effect of drying techniques on the antioxidant properties of *bignay* pomace

Three common assays were used to determine the antioxidant properties of *bignay* pomace. Data gathered revealed that freeze-drying had a positive effect on the antioxidant properties of *bignay* pomace as shown in Table 3. The antioxidant capacity of *bignay* pomace consistently increased in all the tests after undergoing freeze-drying process. Using the DPPH assay, the free-radical scavenging activity of *bignay* pomace after freeze-drying increased by 23% while it did not change significantly with convection oven-drying. In terms of FRAP, the activity of convection oven-dried pomace was reduced by 13% while the freeze-dried pomace showed an increase of 7%. The data showed that ferric reducing antioxidant power of *bignay* pomace was significantly influenced by drying. The ABTS scavenging activity of freeze-dried pomace remarkably increased by 36%. Both drying techniques increased the ABTS scavenging activity of *bignay* pomace but the effect of convection oven-drying was not significant.

**Table 3:** Antioxidant properties of *bignay* pomace as affected by drying techniques

Treatment	DPPH Radical Scavenging Activity	Ferric Reducing Antioxidant Power	ABTS Scavenging Activity
**Fresh**	1283.44 ± 101.46^b^	2674.52 ± 140.11^b^	1618.65 ± 24.04^b^
**Convection oven-dried**	1243.06 ± 58.14^b^	2405.72 ± 34.43^c^	1668.54 ± 58.32^b^
**Freeze-dried**	1574.63 ± 48.18^a^	2935.92 ± 57.79^a^	2199.59 ± 45.11^a^

Values are expressed as mean ± standard deviation (*n* = 5). Means with different superscripts (a-c) within a column, indicate significant differences (*p*<0.05) based on ANOVA and Tukey’s HSD test. Values are expressed as mg Trolox equivalent/100 g DW

Certain processing techniques can negatively or positively affect the bioactivity of some antioxidants. Higher antioxidant activity was observed in freeze-dried sample compared to fresh and convection oven-dried samples. Based on several studies, freeze-drying often results in higher antioxidant activities than samples exposed to thermal drying process (Çoklar & Akbulut 2017; Kasunmala et al. 2021; Saifullah et al. 2019; Samoticha et al. 2016). In the case of *bignay* pomace, the strong antioxidant activity of freeze-dried sample may be attributed to the higher amounts of phenolic compounds present compared to other samples. For total anthocyanin, there was a significantly higher concentration that was extracted in freeze-dried sample. The concentrations of total flavonoids and total condensed tannins were also highest among the three samples. Their cumulative amounts contributed greatly to the enhanced antioxidant activity of freeze-dried samples as compared to the fresh pomace. On the other hand, subjecting pomace to heat with prolonged exposure to air is more favorable to oxidation and degradation reactions that often results in loss of bioactive components (Silva-Espinoza et al. 2020) as manifested in the convection oven-dried sample. It had the lowest concentration of the phytochemical components measured except for condensed tannins. Hence, it exhibited lower antioxidant activity compared to fresh and freeze-dried samples. Furthermore, subjecting the pomace to elevated temperature could have caused certain polyphenols to be transformed into other compounds that have weak antioxidant potential (Akowuah et al. 2009).

### Effect of drying on the phenolic compound profile and concentration of *bignay* pomace

From the 12 phenolic standards used, nine phenolic compounds were detected in *bignay* pomace using HPLC-PDA and they were identified as catechin, epicatechin, rutin hydrate, syringic acid, ellagic acid, trans-ferulic acid, myricetin, resveratrol, and quercetin (Table 4). All of these phenolic compounds, except syringic acid, were also identified in the analyzed *bignay* fruit samples from different cultivars in a previous study conducted by Butkhup and Samappito (2008). Aside from these, they also identified procyanidin, gallic acid, caffeic acid, naringenin and kaempferol.

**Table 4:** Individual phenolic compounds in fresh and dried *bignay* pomace samples from HPLC analysis

Phenolic Compound	Conc. (mg/100 g DW)		
	Fresh	Convection oven-dried	Freeze-Dried
Catechin	26.97 ± 0.65^a^	20.31 ± 1.81^b^	21.36 ± 0.54^b^
Epicatechin	49.22 ± 2.03^a^	38.62 ± 2.35^b^	40.31 ± 3.61^b^
Rutin hydrate	3.17 ± 0.29^a^	2.44 ± 0.09^b^	2.68 ± 0.17^b^
Syringic acid	3.78 ± 0.13^a^	2.28 ± 0.04^b^	2.28 ± 0.04^b^
Ellagic acid	23.98 ± 0.46^a^	12.57 ± 0.43^b^	13.05 ± 0.56^b^
Trans-ferulic acid	4.70 ± 0.13^a^	2.34 ± 0.09^b^	2.25 ± 0.05^b^
Myricetin	3.97 ± 0.11^a^	2.06 ± 0.02^b^	2.09 ± 0.15^b^
Resveratrol	5.31 ± 0.35^a^	2.98 ± 0.07^b^	2.77 ± 0.08^b^
Quercetin	7.86 ± 0.27^a^	4.04 ± 0.11^c^	4.59 ± 0.07^b^

Values are expressed as mean ± standard deviation (*n* = 3). Means with different superscripts (a-c) within a row, indicate significant differences (*p*<0.05) based on ANOVA and Tukey’s HSD test

Results revealed that *bignay* pomace in its fresh form had the highest concentration of each phenolic compound identified but was significantly reduced after freeze-drying and convection oven-drying (15 to 52%). Drying may lead to degradation of phenolic components because their structures are easily destroyed when subjected or exposed to heat, oxygen and freezing (Tan et al. 2019). A similar result was achieved in red raspberry (*Rubus lambertianus*) where a decrease in the concentration of individual phenolic compounds including catechin, rutin, and quercetin was observed after freeze-drying and hot air-drying (Yu et al. 2018). In the same manner, *Centella asiatica* also exhibited degradation of flavonoid components by up to 97% as an effect of freeze-drying and oven-drying (Mohd Zainol et al. 2009). The drying process may have facilitated the activity of oxidative enzymes such as polyphenol oxidase and peroxidase, which consequently caused the loss of phenolic complexes (Roslan et al. 2020). According to Kumar et al. (2022), for any drying method, there is a disintegration of cellular structures. This makes the phenolic compounds that are outside the organelles to be more prone to degradation (Kumar et al. 2022; Turgay & Esen 2020).

Epicatechin was the most abundant in the fresh, freeze-dried, and convection oven-dried *bignay* pomace amounting to as high as around 50 mg/100 g DW followed by catechin. Ellagic acid was the third most abundant phenolic compound with a concentration of around 13% in the dried samples. The rest of the phenolic compounds were available at a concentration lower than 10 mg/100 g. Epicatechin, catechin, resveratrol, myricetin, quercetin, and ferulic acid were also some of the phenolic constituents identified in the fruit from different cultivars of *bignay* in Thailand by Jorjong et al. (2015). The concentrations of most of the phenolic compounds in the pomace were found to fall within the reported range of Jorjong et al. except for epicatechin and resveratrol. These two compounds were present in lower amounts in pomace compared to the whole fruit.

There was no significant difference found in the concentrations of all the phenolic compounds in the freeze-dried and convection oven-dried pomace except quercetin. Among all the detected phenolic compounds, epicatechin, catechin, and rutin were found to be least affected by both convection oven-drying and freeze-drying, as suggested by the lower percentage reduction in their concentrations (around 15–25%). Using different drying treatments, catechin and rutin were also observed as the most stable in *C. asiatica* (Mohd Zainol et al. 2009). Likewise, rutin according to Frutos et al. (2019), is a generally stable phenolic compounds even when subjected to heat processing. The rest of the phenolic compounds present in the dried pomace exhibited lesser retention. For trans-ferulic acid, the amount was reduced by half after undergoing convection oven and freeze-drying whereas myricetin, resveratrol, ellagic acid, syringic acid, and quercetin had reduction of 40–49%. The different effects of drying on the individual phenolic compound according to Mohd Zainol et al. (2009) is possibly due to variability in the number and arrangement of hydroxyl groups.

## Conclusion

Drying had a significant impact on the physico-chemical and bioactive properties of *bignay* pomace. Both the convection oven- and freeze-drying methods were effective in reducing the moisture content and water activity of the material as well as in enhancing its color characteristics. In terms of bioactive profile and antioxidant properties, the drying process had a varied effect on *bignay* pomace, depending on the specific method applied. Compared to convection oven-drying, freeze-drying resulted in minimal degradation of the total phenolic content whereas the total anthocyanin, total flavonoid, and condensed tannins content increased. This promoted higher antioxidant activity with freeze-drying. Overall, freeze-drying was found to be the suitable drying method for *bignay* pomace to preserve its bioactive properties.

## Data Availability

All data supporting this study are included in this manuscript.

## References

[CR1] Akowuah, G. A., Mariam, A., & Chin, J. H. (2009). The effect of extraction temperature on total phenols and antioxidant activity of Gynura procumbens leaf. *Pharmacognosy Magazine*, *5*(17), 81–85 http://www.phcog.com/text.asp?2009/5/17/81/57991. Accessed 11 Jan 2022.

[CR2] Albuquerque, B. R., Heleno, S. A., Oliveira, M. B., Barros, L., & Ferreira, I. C. (2021). Phenolic compounds: Current industrial applications, limitations and future challenges. *Food Function*, *12*(1), 14–29. 10.1039/d0fo02324h. 33242057 10.1039/d0fo02324h

[CR3] Amaya, E., & Nickell, D. (2015). Using feed to enhance the color quality of fish and crustaceans. In D. A. Davis (Ed.), *Feed and feeding practices in aquaculture*, (pp. 269–298). Woodhead Publishing. 10.1016/B978-0-08-100506-4.00011-8.

[CR4] AOAC (Ed.) (2019). *Official methods of analysis of the Association of Official Analytical Chemists: Official methods of analysis of AOAC international*, (21st ed., ). Washington DC: AOAC.

[CR5] Barcelo, J. M., Nullar, A. M., Caranto, J. P., Gatchallan, A. M., & Aquino, I. B. (2016). Antioxidant and antimutagenic activities of ripe Bignay (*Antidesma bunius*) crude fruit extract. *Philippine e-Journal for Applied Research and Development*, *6*, 32–43.

[CR6] Butkhup, L., & Samappito, S. (2008). Analysis of anthocyanin, flavonoids, and phenolic acids in tropical *bignay* berries. *International Journal of Fruit Science*, *8*(1–2), 15–34. 10.1080/1553836080236591.

[CR7] Capanoglu, E., Nemli, E., & Tomas-Barberan, F. (2022). Novel approaches in the valorization of agricultural wastes and their applications. *Journal of Agricultural and Food Chemistry*, *70*(23), 6787–6804. 10.1021/acs.jafc.1c07104. 35195402 10.1021/acs.jafc.1c07104 PMC9204820

[CR8] Castillo-Israel, K., Sartagoda, K., Illano, M., Flandez, L., Compendio, M., & Morales, D. (2020). Antioxidant properties of Philippine *bignay* (*Antidesma bunius* (Linn.) Spreng CV. ‘Common’) flesh and seeds as affected by fruit maturity and heat treatment. *Food Research*, *4*(6), 1980–1987. 10.26656/fr.2017.4(6).215.

[CR9] Chaaban, H., Ioannou, I., Chebil, L., Slimane, M., Gérardin, C., Paris, C., Charbonnel, C., Chekir, L., & Ghoul, M. (2017). Effect of heat processing on thermal stability and antioxidant activity of six flavonoids. *Journal of Food Processing and Preservation*, *41*(5), e13203. 10.1111/jfpp.13203.

[CR10] Çoklar, H., & Akbulut, M. (2017). Effect of sun, oven and freeze-drying on anthocyanins, phenolic compounds and antioxidant activity of black grape (Ekşikara) (*Vitis vinifera* L.). *South African Journal of Enology and Viticulture*, *38*(2), 264–272. 10.21548/38-2-2127.

[CR11] Ebrahimi, P., & Lante, A. (2021). Polyphenols: A comprehensive review of their nutritional properties. *The Open Biotechnology Journal*, *15*(1), 164–172. 10.2174/1874070702115010164.

[CR12] Elshaafi, I., Musa, K., & Abdullah Sani, N. (2020). Effect of oven and freeze drying on antioxidant activity, total phenolic and total flavonoid contents of fig (*Ficus carica* L.) leaves. *Food Research*, *4*(6), 2114–2121. 10.26656/fr.2017.4(6).072.

[CR13] Fattahi, S., Zabihi, E., Abedian, Z., Pourbagher, R., Motevalizadeh Ardekani, A., Mostafazadeh, A., & Akhavan-Niaki, H. (2014). Total phenolic and flavonoid contents of aqueous extract of stinging nettle and *in vitro* antiproliferative effect on Hela and BT-474 cell lines. *International Journal Of Molecular And Cellular Medicine*, *3*(2), 102–107. PMID: 25035860; PMCID: PMC4082812. PMC4082812 25035860

[CR14] Frutos, M. J., Rincon-Frutos, L., & Valero-Cases, E. (2019). Rutin. In S. M. Nabavi, & A. S. Silva (Eds.), *Nonvitamin and nonmineral nutritional supplements*, (pp. 111–117). Academic Press. 10.1016/B978-0-12-812491-8.00015-1.

[CR15] Gao, Q., Wu, C., Wang, M., Xu, B., & Du, L. (2012). Effect of drying of jujubes (*Ziziphus jujuba* mill.) on the contents of sugars, organic acids, α-tocopherol, β-carotene, and phenolic compounds. *Journal of Agricultural and Food Chemistry*, *60*(38), 9642–9648. 10.1021/jf3026524. 22958080 10.1021/jf3026524

[CR16] Garcìa, L. M., Ceccanti, C., Negro, C., De Bellis, L., Incrocci, L., Pardossi, A., & Guidi, L. (2021). Effect of drying methods on phenolic compounds and antioxidant activity of *Urtica dioica* L. Leaves. *Horticulturae*, *7*(1), 10. 10.3390/horticulturae7010010.

[CR17] Global Agricultural Information Network (2018). *Philippines: US wines maintain stronghold in Philippine market*. USDA Foreign Agricultural Services. USDA Foreign Agricultural Services https://apps.fas.usda.gov/newgainapi/api/report/downloadreportbyfilename?filename=US%20Wines%20Maintain%20Stronghold%20in%20Philippine%20Market_Manila_Philippines_4-20-2018.pdf.

[CR18] Gümüşay, Ö. A., & Yalçın, M. Y. (2019). Effects of freeze-drying process on antioxidant and some physical properties of cherry laurel and kiwi fruits. *Academic Food Journal / Akademik GIDA*, *17*(1), 9–15. 10.24323/akademik-gida.543985.

[CR19] Hardinasinta, G., Mursalim, M., Muhidong, J., & Salengke, S. (2020). Determination of some chemical compounds of *bignay* (*Antidesma bunius*) fruit juice. *Food Science and Technology*, *41*(4), 974–979. 10.1590/fst.27720.

[CR20] Iqbal, A., Schulz, P., & Rizvi, S. S. (2021). Valorization of bioactive compounds in fruit pomace from agro-fruit industries: Present insights and future challenges. *Food Bioscience*, *44*, 101384. 10.1016/j.fbio.2021.101384.

[CR21] Islary, A., Sarmah, J., & Basumatary, S. (2017). Nutritional value, phytochemicals and antioxidant properties of two wild edible fruits (*Eugenia operculate* Roxb. and *Antidesma bunius* L.) from Assam, North-East India. *Mediterranean Journal of Nutrition and Metabolism*, *10*(1), 29–40. 10.3233/MNM-16119.

[CR22] ISO (2005). The International Organization for Standardization, 14502–1. Determination of substances characteristic of black and green tea, Part 1: Determination of total polyphenols in tea – Colorimetric method using Folin-Ciocalteu reagent.

[CR23] Izli, N., Izli, G., & Taskin, O. (2018). Impact of different drying methods on the drying kinetics, color, total phenolic content and antioxidant capacity of pineapple. *CyTA – Journal of Food*, *16*(1), 213–221. 10.1080/19476337.2017.1381174.

[CR24] Jorjong, S., Butkhup, L., & Samappito, S. (2015). Phytochemicals and antioxidant capacities of Mao-Luang (*Antidesma bunius* L.) cultivars from northeastern Thailand. *Food Chemistry*, *181*, 248–255. 10.1016/j.foodchem.2015.02.093. 25794747 10.1016/j.foodchem.2015.02.093

[CR25] Karma, I. G. (2020). Determination and measurement of color dissimilarity. *International Journal of Engineering and Emerging Technology*, *5*(1), 67–71. 10.24843/ijeet.2020.v05.i01.p13.

[CR26] Kasunmala, I. G. G., Navarathne, S. B., & Wickramasinghe, I. (2021). Effect of drying methods on antioxidant activity of *Syzygium caryophyllatum* (L.) fruit pulp. *International Journal of Fruit Science*, *21*(1), 634–644. 10.1080/15538362.2021.1911744.

[CR27] Kumar, D., Ladaniya, M. S., Gurjar, M., & Kumar, S. (2022). Impact of drying methods on natural antioxidants, phenols and flavanones of immature dropped *Citrus sinensis* L. Osbeck fruits. *Scientific Reports*, *12*(1). 10.1038/s41598-022-10661-7. 10.1038/s41598-022-10661-7 PMC9035179 35461355

[CR28] Kwok, B. H. L., Hu, C., Durance, T., & Kitts, D. D. (2004). Dehydration techniques affect phytochemical contents and free radical scavenging activities of Saskatoon berries (*Amelanchier alnifolia* Nutt.). *Journal of Food Science*, *69*, SNQ122–SNQ126. 10.1111/j.1365-2621.2004.tb13381.x.

[CR29] Larrauri, J. A., Rupérez, P., & Saura-Calixto, F. (1997). Effect of drying temperature on the stability of polyphenols and antioxidant activity of red grape pomace peels. *Journal of Agricultural and Food Chemistry*, *45*(4), 1390–1393. 10.1021/jf960282f.

[CR30] Latos-Brozio, M., & Masek, A. (2020). Natural polymeric compound based on high thermal stability catechin from green tea. *Biomolecules*, *10*(8), 1191. 10.3390/biom10081191. 32824310 10.3390/biom10081191 PMC7464854

[CR31] Marinova, G., & Batchvarov, V. (2011). Evaluation of the methods for determination of the free radical scavenging activity by DPPH. *Bulgarian Journal of Agricultural Science.*, *17*(1), 11–24 https://www.agrojournal.org/17/01-02-11.pdf.

[CR32] Medini, F., Fellah, H., Ksouri, R., & Abdelly, C. (2014). Total phenolic, flavonoid and tannin contents and antioxidant and antimicrobial activities of organic extracts of shoots of the plant *Limonium delicatulum*. *Journal of Taibah University for Science*, *8*(3), 216–224. 10.1016/j.jtusci.2014.01.003.

[CR33] Michalczyk, M., MacUra, R., & Matuszak, I. (2009). The effect of air-drying, freeze-drying and storage on the quality and antioxidant activity of some selected berries. *Jounal of Food Processing and Preservation*, *33*(1), 11–21. 10.1111/j.1745-4549.2008.00232.x.

[CR34] Mohd Zainol, M. K., Abdul-Hamid, A., Abu Bakar, F., & Pak Dek, S. (2009). Effect of different drying methods on the degradation of selected flavonoids in *Centella asiatica*. *International Food Research Journal*, *16*, 531–537.

[CR35] Moritsuka, N., Kawamura, K., Tsujimoto, Y., Rabenarivo, M., Andriamananjara, A., Rakotoson, T., & Razafimbelo, T. (2019). Comparison of visual and instrumental measurements of soil color with different low-cost colorimeters. *Soil Science and Plant Nutrition*, *65*(6), 605–615. 10.1080/00380768.2019.1676624.

[CR36] Mphahlele, R. R., Fawole, O. A., Makunga, N. P., & Opara, U. L. (2016). Effect of drying on the bioactive compounds, antioxidant, antibacterial and antityrosinase activities of pomegranate peel. *BMC Complementary and Alternative Medicine*, *16*(1), 143. 10.1186/s12906-016-1132-y. 27229852 10.1186/s12906-016-1132-y PMC4881059

[CR37] Novasina AG. (2010). LabSwift-aw Operating Instructions [Pamphlet]. https://www.novasina.ch/wp-content/uploads/2021/05/Ba-LabSwift-E-003529_03.pdf. Accessed 12 Sept 2021.

[CR38] Orphanides, A., Goulas, V., & Gekas, V. (2013). Effect of drying method on the phenolic content and antioxidant capacity of spearmint. *Czech Journal of Food Sciences*, *31*(5), 509–513. 10.17221/526/2012-cjfs.

[CR39] Palaniveloo, K., Amran, M. A., Norhashim, N. A., Mohamad-Fauzi, N., Peng-Hui, F., Hui-Wen, L., … Razak, S. A. (2020). Food waste composting and microbial community structure profiling. *Processes*, *8*(6), 723. 10.3390/pr8060723.

[CR40] Pathare, P. B., Opara, U. L., & Al-Said, F. A. J. (2013). Colour measurement and analysis in fresh and processed foods: A review. *Food and Bioprocess Technology*, *6*(1), 36–60. 10.1007/s11947-012-0867-9. 10.1007/s11947-012-0932-4 PMC7089433 32215166

[CR41] Prabhakar, K., & Mallika, E. N. (2014). Dried foods. In C. A. Batt (Ed.), *Encyclopedia of Food Microbiology*, (2nd ed., pp. 574–576). Academic Press.

[CR42] Qu, Q., Yang, X., Fu, M., Chen, Q., Zhang, X., He, Z., & Qiao, X. (2015). Effects of three conventional drying methods on the lipid oxidation, fatty acids composition, and antioxidant activities of walnut (*Juglans regia*L.). *Drying Technology*, *34*(7), 822–829. 10.1080/07373937.2015.1081931.

[CR43] Rahman, N. F., Shamsudin, R., Ismail, A., Shah, N. N., & Varith, J. (2018). Effects of drying methods on total phenolic contents and antioxidant capacity of the pomelo (*Citrus grandis *(L.) Osbeck) peels. *Innovative Food Science & Emerging Technologies*, *50*, 217–225. 10.1016/j.ifset.2018.01.009.

[CR44] Recuenco, M. C., Lacsamana, M. S., Hurtada, W. A., & Sabularse, V. S. (2016). Total phenolic and Total flavonoid contents of selected fruits in the Philippines. *Philippine Journal of Science*, *154*(3), 276–281.

[CR45] Roslan, A. S., Ismail, A., Ando, Y., & Azlan, A. (2020). Effect of drying methods and parameters on the antioxidant properties of tea (*Camellia sinensis*) leaves. *Food Production, Processing and Nutrition*, *2*(1). 10.1186/s43014-020-00022-0.

[CR46] Sablani, S. S., Andrews, P. K., Davies, N. M., Walters, T., Saez, H., & Bastarrachea, L. (2011). Effects of air and freeze drying on phytochemical content of conventional and organic berries. *Drying Technology*, *29*(2). 10.1080/07373937.2010.483047.

[CR47] Saifullah, M., McCullum, R., McCluskey, A., & Vuong, Q. (2019). Effects of different drying methods on extractable phenolic compounds and antioxidant properties from lemon myrtle dried leaves. *Heliyon*, *5*(12), e03044. 10.1016/j.heliyon.2019.e03044. 31890968 10.1016/j.heliyon.2019.e03044 PMC6928250

[CR48] Samoticha, J., Wojdyło, A., & Lech, K. (2016). The influence of different drying methods on chemical composition and antioxidant activity in chokeberries. *LWT*, *66*, 484–489. 10.1016/j.lwt.2015.10.073.

[CR49] Santos, S. S. D., Paraíso, C. M., & Madrona, G. S. (2020). Blackberry pomace microspheres: An approach on anthocyanin degradation. *Ciência e Agrotecnologia*, *44*, 014420. 10.1590/1413-7054202044014420.

[CR50] Seke, F., Manhivi, V. E., Shoko, T., Slabbert, R. M., Sultanbawa, Y., & Sivakumar, D. (2021). Effect of freeze drying and simulated gastrointestinal digestion on phenolic metabolites and antioxidant property of the natal plum (*Carissa macrocarpa*). *Foods*, *10*(6), 1420. 10.3390/foods10061420. 34207411 10.3390/foods10061420 PMC8235007

[CR51] Shariful Islam, M., Sharif Ahammed, M., Islam Sukorno, F., Ferdowsy Koly, S., Morad Biswas, M., & Hossain, S. (2018). A review on phytochemical and pharmacological potentials of *Antidesma bunius*. *Journal of Analytical and Pharmaceutical Research*, *7*(5). 10.15406/japlr.2018.07.00289.

[CR52] Shofian, N. M., Hamid, A. A., Osman, A., Saari, N., Anwar, F., Pak Dek, M. S., & Hairuddin, M. R. (2011). Effect of freeze-drying on the antioxidant compounds and antioxidant activity of selected tropical fruits. *International Journal of Molecular Sciences*, *12*(7), 4678–4692. 10.3390/ijms12074678. 21845104 10.3390/ijms12074678 PMC3155377

[CR53] Silva-Espinoza, M. A., Ayed, C., Foster, T., del Mar Camacho, M., & Martínez-Navarrete, N. (2020). The impact of freeze-drying conditions on the physico-chemical properties and bioactive compounds of a freeze-dried orange puree. *Foods*, *9*(1). 10.3390/foods9010032=. 10.3390/foods9010032 PMC7022254 31905861

[CR54] Singhal, P., Satya, S., Naik, N., & S. (2022). Effect of different drying techniques on the nutritional, antioxidant and cyanogenic profile of bamboo shoots. *Applied Food Research*, *2*(1), 100036. 10.1016/j.afres.2021.100036.

[CR55] Sokač, T., Gunjević, V., Pušek, A., Tušek, A. J., Dujmić, F., Brnčić, M., … Redovniković, I. R. (2022). Comparison of drying methods and their effect on the stability of Graševina grape pomace biologically active compounds. *Foods*, *11*(1), 112. 10.3390/foods11010112. 35010238 10.3390/foods11010112 PMC8750427

[CR56] Tan, S., Tang, J., Shi, W., Wang, Z., Xiang, Y., Deng, T., Gao, X., Li, W., & Shi, S. (2019). Effects of three drying methods on polyphenol composition and antioxidant activities of *Litchi chinensis *Sonn. *Food Science and Biotechnology*, *29*(3), 351–358. 10.1007/s10068-019-00674-w. 32257518 10.1007/s10068-019-00674-w PMC7105535

[CR57] Tao, Y., Wu, D., Zhang, Q., & Sun, D. (2014). Ultrasound-assisted extraction of phenolics from wine lees: Modeling, optimization and stability of extracts during storage. *Ultrasonics Sonochemistry*, *21*(2), 706–715. 10.1016/j.ultsonch.2013.09.005. 24090833 10.1016/j.ultsonch.2013.09.005

[CR58] Tapia, M. S., Alzamora, S. M., & Chirife, J. (2020). Effects of water activity (aw) on microbial stability as a hurdle in food preservation. In G. V. Barbosa-Cánovas, J. Anthony, J. Fontana, S. J. Schmidt, & T. P. Labuza (Eds.), *Water activity in foods: Fundamentals and applications*, (pp. 323–355). Wiley. 10.1002/9781118765982.ch14.

[CR59] Tomasina, F., Carabio, C., Celano, L., & Thomson, L. (2012). Analysis of two methods to evaluate antioxidants. *Biochemistry and Molecular Biology Education*, *40*(4), 266–270. 10.1002/bmb.20617. 22807430 10.1002/bmb.20617

[CR60] Tseng, A., & Zhao, Y. (2012). Effect of different drying methods and storage time on the retention of bioactive compounds and antibacterial activity of wine grape pomace (pinot noir and merlot). *Journal of Food Science*, *77*(9), H192–H201. 10.1111/j.1750-3841.2012.02840.x. 22908851 10.1111/j.1750-3841.2012.02840.x

[CR61] Turgay, Ö., & Esen, Y. (2020). Antioxidant, total phenolic, ascorbic acid and color changes of *Ocimum bacilicum* L. by sun and microwave drying. *Food and Health*, *6*(2), 110–116. 10.3153/fh20012.

[CR62] Vera Zambrano, M., Dutta, B., Mercer, D. G., MacLean, H. L., & Touchie, M. F. (2019). Assessment of moisture content measurement methods of dried food products in small-scale operations in developing countries: A review. *Trends in Food Science & Technology*, *88*, 484–496. 10.1016/j.tifs.2019.04.006.

[CR63] Yu, J., Shangguan, Z., Yang, X., Sun, D., Zhu, B., & Ouyang, J. (2018). Effect of drying on the bioactive compounds and antioxidant activity of *Rubus lambertianus*. *International Journal of Food Engineering*, *14*(2). 10.1515/ijfe-2016-0412.

